# C-Reactive Protein-Based Strategy to Reduce Antibiotic Dosing for the Treatment of Pneumococcal Infection

**DOI:** 10.3389/fimmu.2020.620784

**Published:** 2021-01-20

**Authors:** Donald N. Ngwa, Sanjay K. Singh, Alok Agrawal

**Affiliations:** Department of Biomedical Sciences, James H. Quillen College of Medicine, East Tennessee State University, Johnson City, TN, United States

**Keywords:** C-reactive protein, clarithromycin, pneumococcal infection, *Streptococcus pneumoniae*, combination therapy

## Abstract

C-reactive protein (CRP) is a component of innate immunity. The concentration of CRP in serum increases in microbial infections including *Streptococcus pneumoniae* infection. Employing a mouse model of pneumococcal infection, it has been shown that passively administered human wild-type CRP protects mice against infection, provided that CRP is injected into mice within two hours of administering pneumococci. Engineered CRP (E-CRP) molecules have been reported recently; unlike wild-type CRP, passively administered E-CRP protected mice against infection even when E-CRP was injected into mice after twelve hours of administering pneumococci. The current study was aimed at comparing the protective capacity of E-CRP with that of an antibiotic clarithromycin. We established a mouse model of pneumococcal infection in which both E-CRP and clarithromycin, when used alone, provided minimal but equal protection against infection. In this model, the combination of E-CRP and clarithromycin drastically reduced bacteremia and increased survival of mice when compared to the protective effects of either E-CRP or clarithromycin alone. E-CRP was more effective in reducing bacteremia in mice treated with clarithromycin than in untreated mice. Also, there was 90% reduction in antibiotic dosing by including E-CRP in the antibiotic-treatment for maximal protection of infected mice. These findings provide an example of cooperation between the innate immune system and molecules that prevent multiplication of bacteria, and that should be exploited to develop novel combination therapies for infections against multidrug-resistant pneumococci. The reduction in antibiotic dosing by including E-CRP in the combination therapy might also resolve the problem of developing antibiotic resistance.

## Introduction

C-reactive protein (CRP) is a critical host defense molecule of the innate immune system (1, 2). CRP binds to cells and molecules, host or foreign, which have accessible phosphocholine (PCh) moieties (3, 4). One example of CRP-ligands is pneumococcal C-polysaccharide found on the surface of *Streptococcus pneumoniae* (5). Once bound to a PCh-bearing ligand, CRP activates the complement system in both human and murine sera to damage and, if possible, eliminate the ligand (6–9). Experiments employing human CRP transgenic mice, CRP-deficient mice, and normal mice in which human wild-type CRP (WT CRP) was passively administered have all revealed that CRP is protective against pneumococcal infection (2, 10–15). It has also been shown that the anti-pneumococcal activity of CRP *in vivo* is due to the ability of ligand-complexed CRP to activate the complement system (9, 16–18). However, WT CRP was found to be protective only when given to mice within 2 h of administering pneumococci in the mouse model of infection in which WT CRP is passively administered (16).

CRP is a pentameric molecule comprised of five identical subunits (19, 20). Recently, two types of engineered pentameric CRP (E-CRP), E-CRP-1, and E-CRP-2, generated by oligonucleotide-directed site-specific mutagenesis of WT CRP cDNA, have been reported (21). In E-CRP-1, four amino acid residues were mutated (E42Q/F66A/T76Y/E81A). In E-CRP-2, two amino acid residues were mutated (Y40F/E42Q). Tyr^40^ and Glu^42^ are present in the intrinsically disordered region of CRP. Glu^42^ is also a part of the inter-subunit contact region. Phe^66^, Thr^76^ and Glu^81^ form the PCh-binding site of CRP (19, 20). E-CRP-1 does not bind to PCh while E-CRP-2, like WT CRP, binds to PCh. Both E-CRP-1 and E-CRP-2 bind to complement inhibitor factor H recruited by pneumococci on their surface in mouse circulation; WT CRP does not bind to immobilized factor H (22–25). Unlike passively administered WT CRP, both E-CRP-1 and E-CRP-2 protected mice against infection even when E-CRP was administered 12 h after administering pneumococci, indicating that a conformationally altered form of CRP that can bind to immobilized factor H is required for CRP-mediated protection of mice against late-stage pneumococcal infection (1, 21). Accordingly, it was hypothesized that in individuals in whom the conformation of CRP remains unchanged, perhaps due to inappropriate inflammatory conditions around CRP, CRP is not fully functional during infection (21). It was proposed that the use of E-CRP might be beneficial for treatment of infections against antibiotic-resistant pneumococci (21).

Antibiotics are commonly used to treat pneumococcal infection in humans (26). The aim of this study was to directly compare the protective effects (increase in survival and decrease in bacteremia) of E-CRP and antibiotics. Since the antibiotic clarithromycin has been used previously in mouse models of pneumococcal infection and was found to be protective when administered into mice later during the infection (27), clarithromycin was selected for the current study. Both E-CRP-1 and E-CRP-2 were included in the study to compare the protective effects with that of clarithromycin. A mouse model of pneumococcal infection was employed in which E-CRP-1, E-CRP-2, and clarithromycin, when used singly, provided minimal but equal protection against infection. This model was suitable to investigate the protection against infection when E-CRP-1 or E-CRP-2 and clarithromycin were combined for the treatment of mice. The results of the experiments indicate that E-CRP is more effective in reducing bacteremia in mice when used in the presence of clarithromycin.

## Materials and Methods

### Preparation of CRP

The cDNAs for E-CRP-1 (E42Q/F66A/T76Y/E81A mutant CRP) and E-CRP-2 (Y40F/E42Q mutant CRP) were constructed and expressed in CHO cells using the ExpiCHO Expression System (Thermo Fisher Scientific), as described earlier (21). Purification of E-CRP-1 from cell culture supernatants involved Ca^2+^-dependent affinity chromatography on a phosphoethanolamine-conjugated Sepharose column, followed by ion-exchange chromatography on a MonoQ column and gel filtration on a Superose12 column, as described earlier (14). E-CRP-2 was purified by Ca^2+^-dependent affinity chromatography on a PCh-conjugated Sepharose column, followed by ion-exchange chromatography on a MonoQ column and gel filtration on a Superose12 column, as described earlier (8). Native WT CRP was purified from discarded human pleural fluid by Ca^2+^-dependent affinity chromatography on a PCh-conjugated Sepharose column, followed by ion-exchange chromatography on a MonoQ column and gel filtration on a Superose12 column, as described earlier (8), and was used throughout this study. Purified CRP was dialyzed against 10 mM Tris-HCl, pH 7.2, containing 150 mM NaCl and 2 mM CaCl_2_, and was subsequently treated with Detoxi-Gel Endotoxin Removing Gel (Thermo Fisher Scientific) according to manufacturer’s instructions. The concentration of endotoxin in CRP preparations was determined by using the Limulus Amebocyte Lysate kit QCL-1000 (Lonza). Purified CRP was stored at 4°C and used within a week for mouse protection experiments.

### Pneumococci

*Streptococcus pneumoniae* type 3, strain WU2, was obtained as a gift from Dr. David Briles (University of Alabama at Birmingham, Birmingham, AL, USA). Pneumococci were made virulent by sequential i.v. passages in mice and were stored in aliquots at −80°C, as described previously (14, 15). For each experiment, a separate aliquot of pneumococci was thawed and cultured, as described previously (14, 15). Cultured pneumococci were resuspended in normal saline at a concentration of 3.5 × 10^8^ cfu/ml based on the absorbance of the resuspension at 600 nm (A_600_ = 1.00 = 1.2 × 10^9^ cfu/ml). Within 2 h, 100 µl (3.5 × 10^7^ cfu) of pneumococci suspension was injected into each mouse, as reported previously (14, 15, 21). The concentration of pneumococci was confirmed next day by plating on sheep blood agar plates.

### Mice

Male C57BL/6J mice (Jackson Laboratories) were brought up and maintained according to protocols approved by the University Committee on Animal Care. Mice were 8–10 weeks old when used in experiments.

### Determination of the Experimental Dose of Clarithromycin

**Figure 1:**
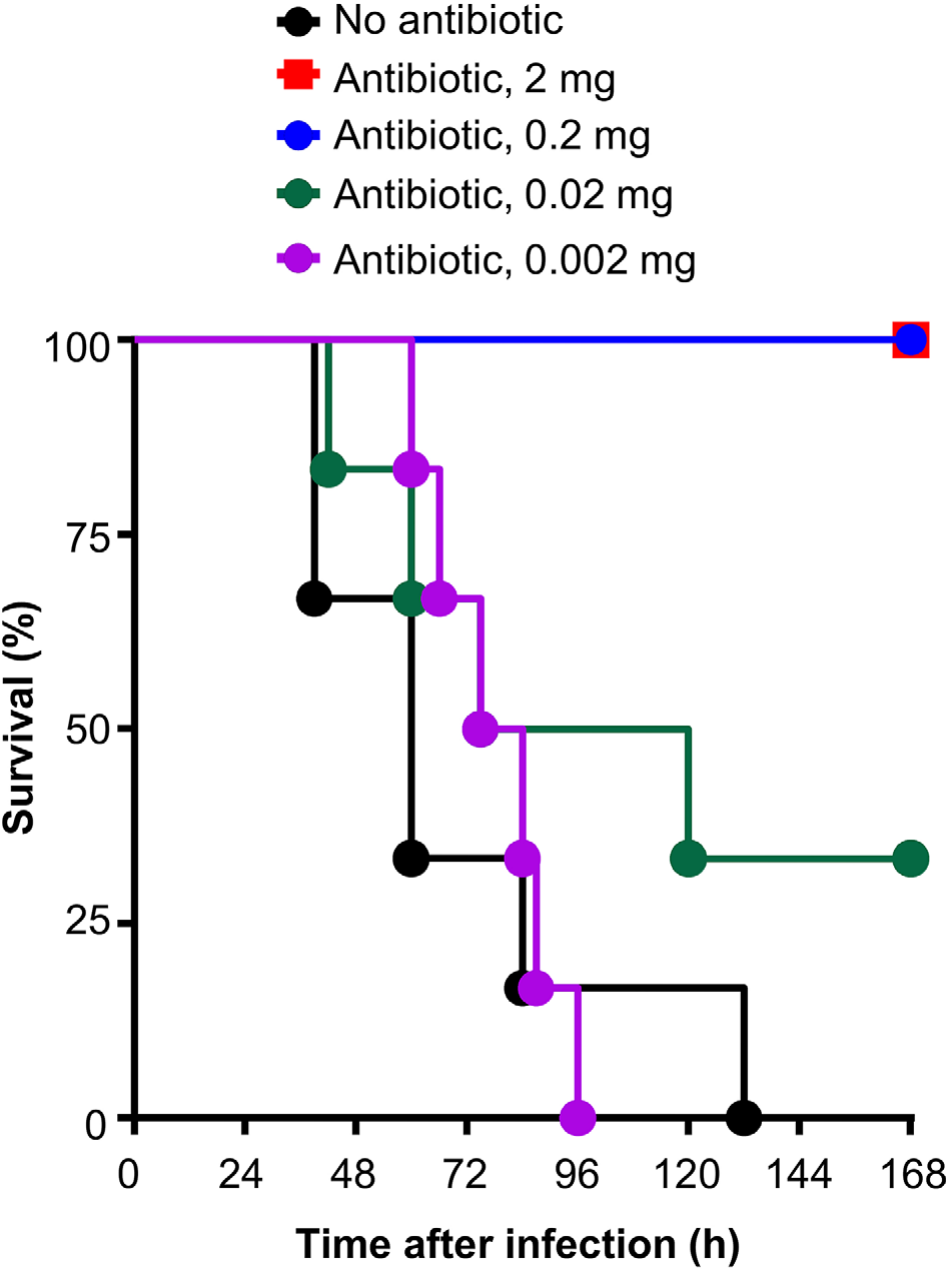
Survival of mice infected with pneumococci and treated with different doses of clarithromycin. Clarithromycin was injected four times, at 12, 36, 60 and 84 h, after the administration of pneumococci. Six mice were used for each dose of clarithromycin.

The antibiotic clarithromycin (Santa Cruz Biotechnology, sc-205634) was reconstituted in acetone at a concentration of 50 mg/ml and stored at 4°C for a maximum of 5 days. To determine the experimental dose for *in vivo* experiments, clarithromycin (50 mg/ml) was diluted in acetone at 40, 4.0, 0.4 and 0.04 mg/ml, and 50 µl of each concentration was injected i.v. into mouse, four times, at 12, 36, 60 and 84 h after the administration of pneumococci. Thus, the final amount of clarithromycin in each group of mice was 2, 0.2, 0.02 and 0.002 mg per mouse per injection. As shown in **Figure 1**, it was clear that a dose of 0.02 mg clarithromycin per mouse per injection was most suitable to evaluate the effects of the combination of clarithromycin and E-CRP on the protection of mice against infection. The dose of 0.02 mg of clarithromycin resulted in a survival curve which fell in the middle so that a shift of the curve, either above or below, could be observed when E-CRP is added.

### Mouse Protection Experiments

Two separate protection experiments were performed using 25 μg of purified WT CRP, E-CRP-1 and E-CRP-2, and 3.5 × 10^7^ cfu of pneumococci, as described earlier (14, 15, 21). The average amount of endotoxin in 25 μg of all CRP preparations was 1.2±1.1 endotoxin units. Mice were first injected i.v. with 3.5 × 10^7^ cfu of pneumococci. The actual number of pneumococci injected, based on the plating results obtained on the next day, was 3.55 ± 0.44 × 10^7^ cfu. Mice were injected i.v. with either WT CRP, E-CRP-1 or E-CRP-2, 12 h after the administration of pneumococci. Since clarithromycin is soluble in nornal saline at 0.2 mg/ml, stock clarithromycin (50 mg/ml in acetone) was diluted in normal saline to a final concentration of 0.2 mg/ml, and 100 µl was injected i.v. per mouse at 13, 36, 60 and 84 h after the administration of pneumococci, to achieve a final dose of 0.02 mg of clarithromycin per mouse per injection. Survival of mice was recorded three times per day for 7 days. To determine bacteremia (cfu/ml) in the surviving mice, blood was collected daily for 5 days from the tip of the tail vein, diluted in normal saline, and plated on sheep blood agar for colony counting. The bacteremia value for dead mice was recorded as 10^9^ cfu/ml because mice died when the bacteremia exceeded 10^8^ cfu/ml.

### Statistical Analysis

Survival curves were generated using the GraphPad Prism 4 software. To determine *p*-values for the differences in the survival curves among various groups, the survival curves were compared using the software’s Logrank (Mantel-Cox) test. The scatter plots of the bacteremia data and the median bacteremia value for each group were generated using the GraphPad Prism 4 software. Bacteremia values of 0–100 were plotted as 100 and bacteremia values of >10^8^ were plotted as 10^9^. To determine *p*-values for the differences in bacteremia among various groups at each time point, scatter plots were compared using the software’s Mann-Whitney test. The software’s Mann-Whitney test included all the dots in the scatter plots and not just the median values for each time point.

## Results

### Anti-Pneumococcal Effects of E-CRP-1, Clarithromycin, and Their Combination

**Figure 2:**
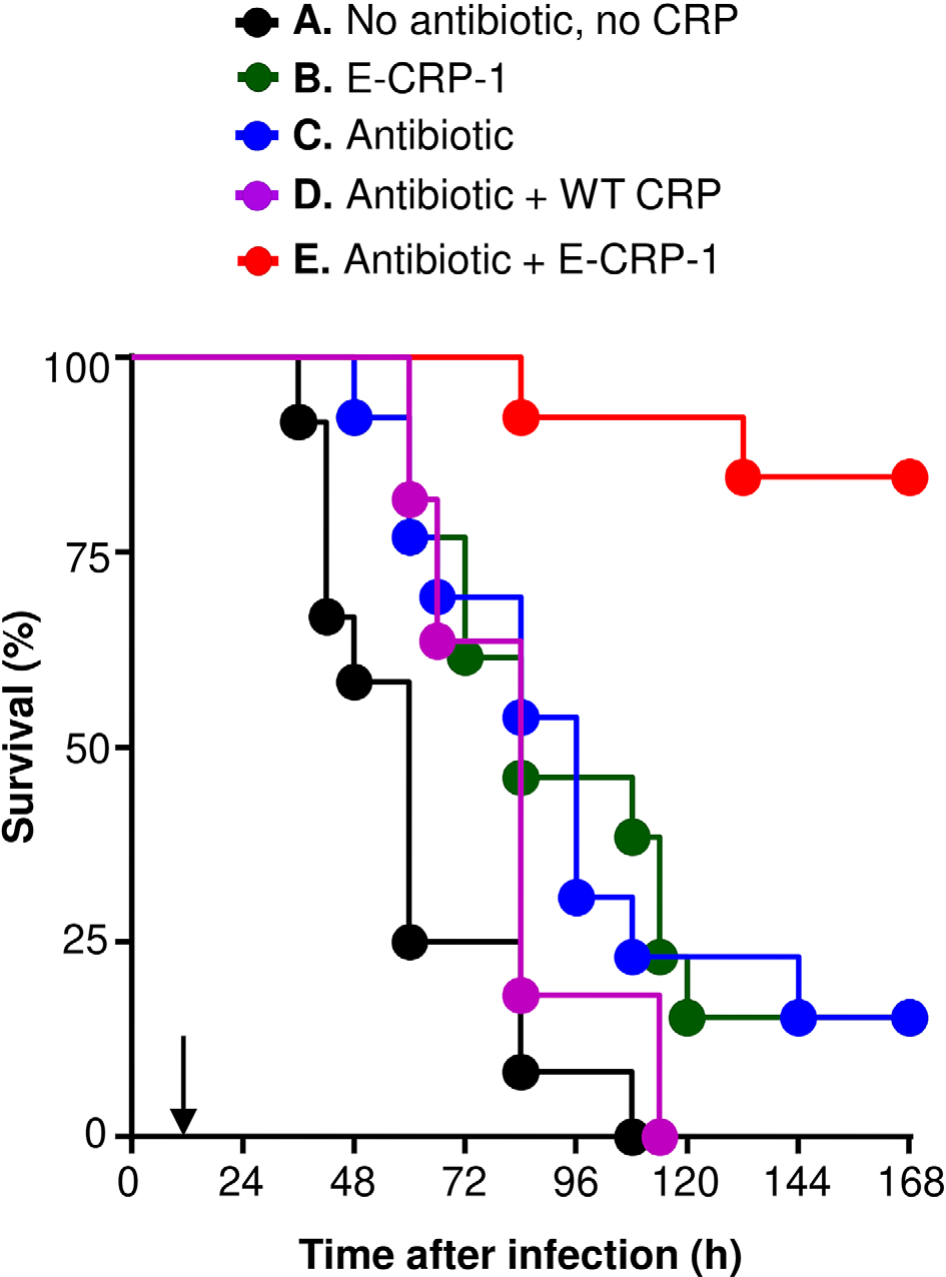
Survival of mice infected with pneumococci and treated with E-CRP-1 and clarithromycin. E-CRP-1 or WT CRP was injected 12 h after administering pneumococci and is indicated by an arrow on the x-axis. Clarithromycin (0.02 mg) was injected four times, at 13, 36, 60 and 84 h, after the administration of pneumococci. The data are combined from two separate experiments with six to eight mice in each group in each experiment. The *p*-values for the differences in the survival curves between groups A B and A C were 0.004 and 0.006, respectively. The *p*-value for the difference in the survival curves between groups B and C was 0.94. The *p*-values for the differences in the survival curves between groups C D and C E were 0.23 and <0.001, respectively. The *p*-values for the differences in the survival curves between groups B E and D E were <0.001.

As shown in **Figure 2**, and as reported previously (21), E-CRP-1 increased significantly the median survival time (MST, the time taken for the death of 50% of mice) of mice infected with pneumococci. The MST for mice injected with bacteria alone (group A) was 60 h while the MST for mice injected with E-CRP-1 (group B) was 84 h. Similarly, clarithromycin also significantly increased the survival of mice infected with pneumococci; the MST for mice treated with clarithromycin (group C) was 96 h. The increase in the MST for mice treated with either E-CRP-1 or clarithromycin were not significantly different from each other. In this mouse model of pneumococcal infection, it has been reported previously that WT CRP does not increase the MST if mice received WT CRP 12 h after receiving pneumococci (21). The protection in response to the combination of WT CRP and clarithromycin (group D) was similar to that of clarithromycin alone. The MST for mice treated with the combination of WT CRP and clarithromycin was 84 h similar to the MST for mice treated with clarithromycin alone (group C). In contrast to WT CRP, the combination of E-CRP-1 and clarithromycin significantly increased the survival of infected mice (group E). The MST for mice treated with both E-CRP-1 and clarithromycin was significantly different than the MST for mice treated with either agent alone. The MST for mice treated with E-CRP-1 and clarithromycin could not be calculated since ~90% of mice survived till the end of the experiment. In all other groups, >75% mice died within 5 days. The combination of E-CRP-1 and 0.02 mg clarithromycin provided the same protection (**Figure 2**) in terms of survival of mice as was seen with 0.2 mg of clarithromycin when used alone (**Figure 1**). Thus, there was 90% reduction in the dose of clarithromycin when used in combination with E-CRP-1.

**Figure 3:**
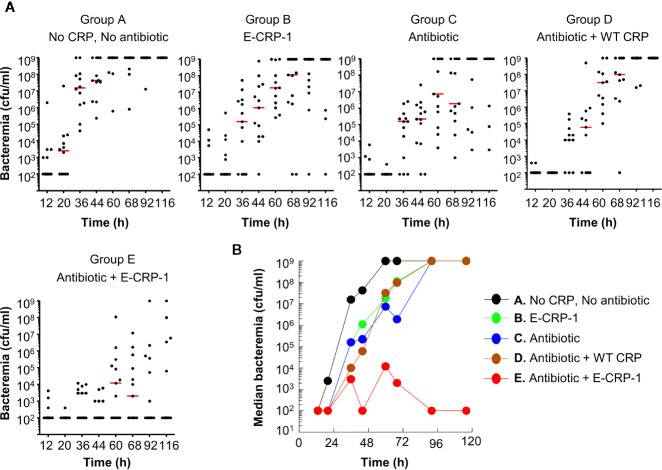
Bacteremia in mice infected with pneumococci and treated with E-CRP-1 and clarithromycin. Blood was collected from each surviving mouse shown in **Figure 2**. **(A)** Scatter plots of the bacteremia data. The horizontal red line in each group of mice represents median bacteremia. **(B)** The median bacteremia values, derived from **(A)** For 36–92 h, the *p*-value for the difference between groups A and B was <0.05. For 20–68 h, the *p*-value for the difference between groups A and C was <0.05. For all time points, the *p*-values for the differences between groups B C and C D were >0.05. For 36–116 h, the *p*-values for the differences between groups C E, B E, and D E were <0.05, with most significant difference (*p* < 0.005) between 44–92 h.

The protective effects of E-CRP-1 (group B) and clarithromycin (group C) on the survival of mice, when used alone, were due to significant decrease in bacteremia (**Figures 3A, B**). E-CRP-1, as reported previously (21), and clarithromycin, both significantly decreased bacteremia. The decrease in bacteremia by E-CRP-1 and by clarithromycin were not significantly different from each other. Consistent with the survival data, the combination of WT CRP and clarithromycin (group D) did not significantly affect the protective ability of clarithromycin in terms of decreasing bacteremia. The dramatic increase in the survival of infected mice by the combination of E-CRP-1 and clarithromycin (group E) was due to the drastic decrease in bacteremia. The decrease in bacteremia in mice treated with both agents was significantly different from the decrease in bacteremia when mice were treated with either of the two agents alone. Bacteremia did not rise beyond 10^4^ cfu/ml in mice treated with both E-CRP-1 and clarithromycin. The median bacteremia was maintained at the reduced level from the beginning to the end of the experiment.

Overall, based on the statistical analyses of the survival curves (**Figure 2**) and of the scatter plots for bacteremia (**Figure 3A**), highly significant differences were found between the groups of mice treated with both E-CRP-1 and clarithromycin and the groups of mice treated with either agent alone. The anti-pneumococcal effects of E-CRP-1 were enhanced in the presence of low-dose clarithromycin. Combining E-CRP-1 with clarithromycin in the treatment of pneumococcal infection in this mouse model reduced the dose of clarithromycin by 90%.

### Anti-Pneumococcal Effects of E-CRP-2, Clarithromycin, and Their Combination

**Figure 4:**
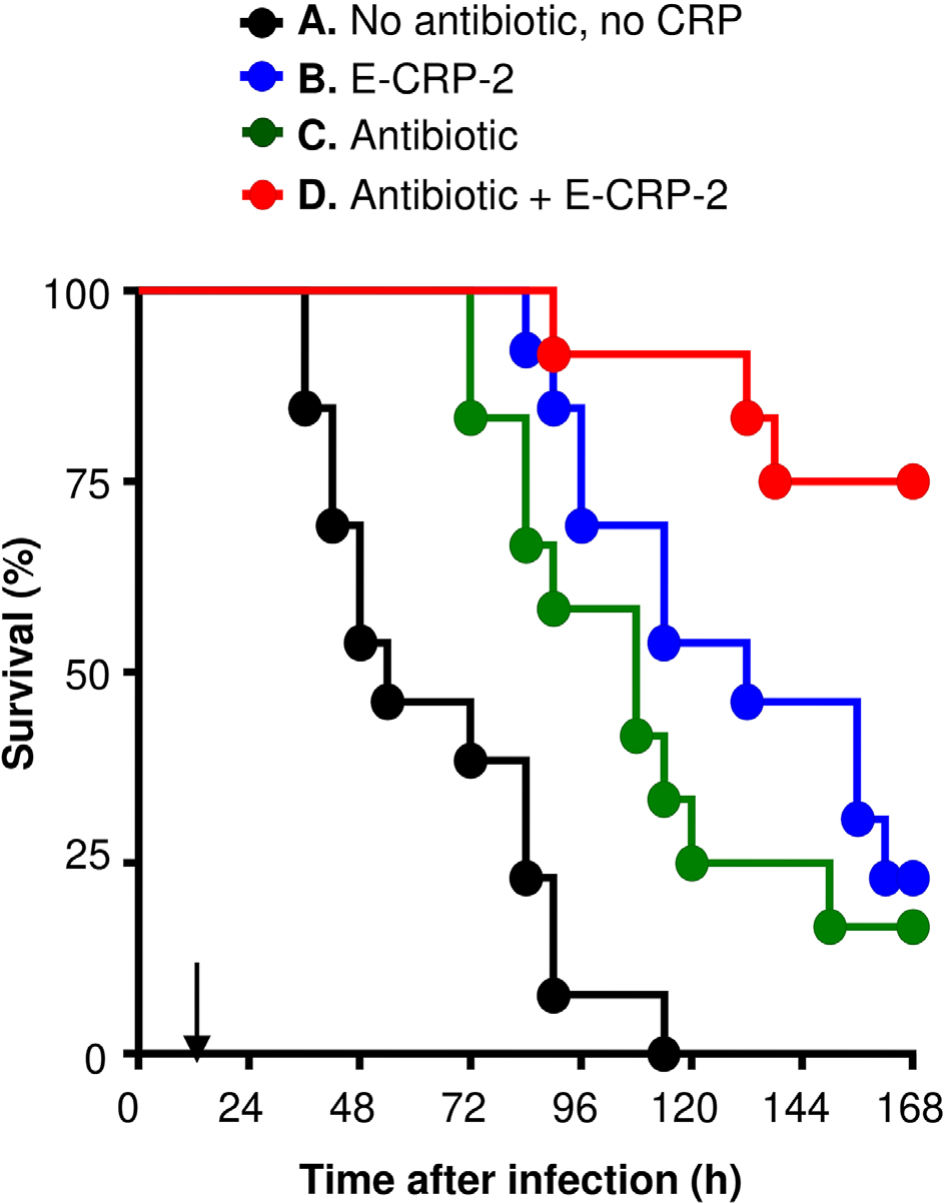
Survival of mice infected with pneumococci and treated with E-CRP-2 and clarithromycin. E-CRP-2 was injected 12 h after administering pneumococci and is indicated by an arrow on the x-axis. Clarithromycin (0.02 mg) was injected four times, at 13, 36, 60 and 84 h, after the administration of pneumococci. The data are combined from two separate experiments with six to eight mice in each group in each experiment. The *p*-values for the differences in the survival curves between groups A B and A C were <0.001 and 0.002, respectively. The *p*-value for the difference in the survival curves between groups B and C was 0.25. The *p*-values for the differences in the survival curves between groups B D and C D were 0.01 and 0.002, respectively.

As shown in **Figure 4**, and as reported previously (21), E-CRP-2 increased significantly the MST of mice infected with pneumococci. The MST for mice injected with bacteria alone (group A) was 54 h while the MST for mice injected with E-CRP-2 (group B) was 132 h. Clarithromycin also significantly increased the survival of mice infected with pneumococci; the MST for mice treated with clarithromycin (group C) was 108 h. The increase in the MST for mice treated with either E-CRP-2 or clarithromycin were not significantly different from each other. The combination of E-CRP-2 and clarithromycin significantly increased the survival of infected mice (group D). The MST for mice treated with both E-CRP-2 and clarithromycin was significantly different than the MST for mice treated with either agent alone. The MST for mice treated with the combination of E-CRP-2 and clarithromycin could not be calculated since ~80% of mice survived till the end of the experiment. In all other groups, >50% mice died in 5 days. The combination of E-CRP-2 and 0.02 mg clarithromycin provided the same protection (**Figure 4**) in terms of survival of mice as was seen with 0.2 mg of clarithromycin when used alone (**Figure 1**). Thus, like E-CRP-1, there was 90% reduction in the dose of clarithromycin when combined with E-CRP-2.

**Figure 5:**
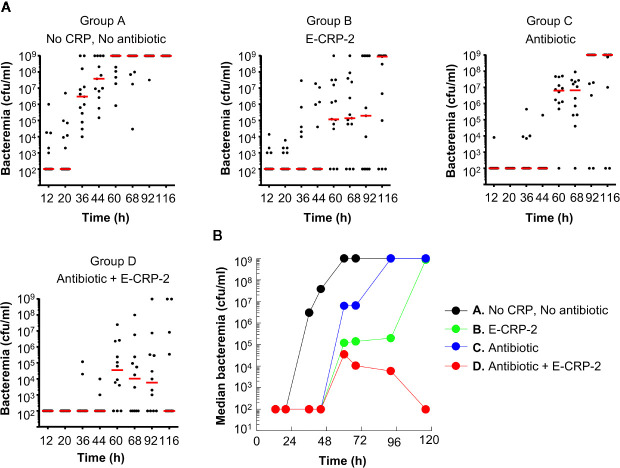
Bacteremia in mice infected with pneumococci and treated with E-CRP-2 and clarithromycin. Blood was collected from each surviving mouse shown in **Figure 4**. **(A)** Scatter plots of the bacteremia data. The horizontal red line in each group of mice represents median bacteremia. **(B)** The median bacteremia values, derived from **(A)** For 36–116 h, the *p*-value for the difference between groups A and B was <0.005. The *p*-values for the difference between groups A and C was <0.005 and <0.05 for 44–68 h and 92–116 h, respectively. For all time points, the *p*-values for the differences between groups B C and B D were >0.05. For 60 to 116 h, the *p*-value for the difference between groups C and D was <0.05.

The protective effects of E-CRP-2 (group B) and clarithromycin (group C) on the survival of mice, when used alone, were due to significant decrease in bacteremia (**Figures 5A, B**). E-CRP-2, as reported previously (21), and clarithromycin, both significantly decreased bacteremia. The decrease in bacteremia by E-CRP-2 and by clarithromycin were not significantly different from each other. The dramatic increase in the survival of infected mice by the combination of E-CRP-2 and clarithromycin (group D) was due to the drastic decrease in bacteremia. The decrease in bacteremia in mice treated with both agents was significantly different from the decrease in bacteremia when mice were treated with either of the two agents alone. Bacteremia did not rise beyond 10^5^ cfu/ml in mice treated with both E-CRP-2 and clarithromycin. The median bacteremia was maintained at the reduced level from the beginning to the end of the experiment.

Overall, based on the statistical analyses of the survival curves (**Figure 4**) and of the scatter plots for bacteremia (**Figure 5A**), highly significant differences were found between the groups of mice treated with the combination of E-CRP-2 and clarithromycin and the groups of mice treated with either agent alone. The anti-pneumococcal effects of E-CRP-2 were enhanced in the presence of low-dose clarithromycin. Combining E-CRP-2 with clarithromycin in the treatment of pneumococcal infection in this mouse model reduced the dose of clarithromycin by 90%.

## Discussion

E-CRP molecules capable of binding to factor H recruited on the surface of pneumococci have been shown to protect mice against late-stage pneumococcal infection (21). Antibiotics protect against pneumococcal infection too (27). The aim of this study was to compare the protective effects of E-CRP with that of an antibiotic clarithromycin employing the same animal model. Our major findings were: 1. Both E-CRP-1 and E-CRP-2, two different molecules capable of binding to factor H recruited on the surface of pneumococci, protected and acted synergistically with clarithromycin to drastically reduce bacteremia and enhance the survival of mice with late-stage pneumococcal infection. 2. The combination of either E-CRP-1 or E-CRP-2 and 0.02 mg clarithromycin provided the same protection as was seen with 0.2 mg of clarithromycin when used alone. There was 90% reduction in the dose of clarithromycin when E-CRP was combined with clarithromycin for the treatment of infected mice. WT CRP did not do so.

Both E-CRP and clarithromycin decrease bacteremia, but via different mechanisms. E-CRP-mediated decrease in bacteremia is due to the activation of the complement system component of innate immunity. Clarithromycin is a macrolide antibiotic that has bacteriostatic action against gram-positive bacteria and some gram-negative bacteria including anaerobes. Clarithromycin is believed to function by binding to the ribosome within the microorganism and inhibiting protein synthesis, and thus inhibiting bacterial growth (26–29). A possible explanation for the synergy between E-CRP and clarithromycin is that the binding of E-CRP to factor H, and perhaps also to other recruited complement inhibitor proteins on pneumococci (21), and subsequent attack by the complement system are more efficient when pneumococci are static; and a low-dose clarithromycin treatment is sufficient to do that. In addition to the bacteriostatic action of clarithromycin, an increasing body of evidence suggests that clarithromycin possesses considerable anti-inflammatory and immunomodulatory properties, such as macrophage activation and inhibition of neutrophilic inflammation (26, 30–32). However, it is not clear whether the anti-inflammatory and immunomodulatory properties of clarithromycin participated in the synergy between clarithromycin and E-CRP in protection against infection. Nevertheless, our data provide a proof of concept that there is cooperation between E-CRP and clarithromycin in reducing bacteremia.

A single dose of E-CRP (25 μg) combined with a tiny amount of clarithromycin (0.02 mg) was the best prescription among all others in this study and in a previously published study (21) for nearly complete protection of our experimental mice. Neither E-CRP (25 μg) nor clarithromycin (0.02 mg) could do it singly, indicating a previously unknown pathway through which the innate immune system responds to antibiotic treatment. The synergy between various CRP species and clarithromycin in reducing bacteremia is just another example of cooperation between a molecule of the innate immune system and antibiotics. It has been reported previously that the classical complement pathway-mediated immunity against antibiotic-resistant pneumococci was enhanced in the presence of sub-inhibitory concentrations of antibiotics cefditoren and ceftriaxone. The binding of CRP to pneumococci was also enhanced in the presence of serum plus either cefditoren or ceftriaxone. Complement activation was also enhanced in the presence of specific anti-pneumococcal antibodies and sub-inhibitory concentrations of antibiotics such as cefditoren, ceftriaxone and amoxicillin (33, 34). It has been suggested that using antibiotics to enhance complement activation might help reduce the impact of antibiotic resistance in pneumococcal infection (33, 34). The cooperation mechanisms between the molecules of the innate immune system and antibiotics should be exploited to develop novel combination therapies to treat infections with antibiotic-resistant pneumococci.

Combination therapies using antimicrobials with different mechanisms of action are used to treat infections against antibiotic-resistant pneumococci and, at the same time, are formulated to prevent the spread of the resistance (35–41). Combination therapies have been shown not only to be more effective against antibiotic-resistant bacteria but also significantly reduce any risk of bacteria developing resistance as seen in monotherapy. The power of E-CRP to reduce antibiotic dosing could be significant and might further assist in these goals to prevent emergence of antibiotic-resistant pneumococci. Usually, combination therapies involve low doses of two antibiotics from different classes. It has been suggested that antibiotic-antibiotic combination therapy may reduce and slow the development of antibiotic resistance. Eliminating one antibiotic from the combination altogether and substituting it with E-CRP should then be, in principle, more effective in reducing and slowing the development of antibiotic resistance. This strategy is similar to antibiotic-non-antibiotic combination therapies, such as adjuvant-antibiotic therapy, where one of the two antibiotics is substituted with an adjuvant with the goal to prevent the development of antibiotic resistance (35–41). We propose that E-CRP should be considered for inclusion in combination therapies. The ability of E-CRP to drastically reduce bacteremia even with a fraction of the normal dose of clarithromycin might contribute further to prevent the development and spread of antibiotic resistance. Our data suggest that, by adding E-CRP to E-CRP-antibiotic combination therapy, the dose of the remaining antibiotic can be kept low.

We conclude that the efficiency of the innate immune system is enhanced in the presence of antibiotics. Our findings provide another example of cooperation between the innate immune system and molecules that prevent multiplication of bacteria, and that should be exploited to develop novel combination therapies for infections against antibiotic-resistant pneumococci. The reduction in antibiotic dosing by the strategy to include E-CRP in the combination therapy using antibiotics might also resolve the problem of developing and spreading antibiotic resistance.

## Data Availability Statement

The original contributions presented in the study are included in the article/supplementary materials, further inquiries can be directed to the corresponding author.

## Ethics Statement

The animal study was reviewed and approved by University Committee on Animal Care, ETSU.

## Author Contributions

DN and SS performed the experiments. AA conceived and designed the experiments. DN, SS, and AA analyzed the data. AA wrote the manuscript with input from DN. All authors contributed to the article and approved the submitted version.

## Funding

This work was supported by the National Institutes of Health grants AR068787, AI117730. and AI151561.

## Conflict of Interest

The authors declare that the research was conducted in the absence of any commercial or financial relationships that could be construed as a potential conflict of interest.

## Abbreviations

CRP, C-reactive protein; E-CRP-1, engineered CRP mutant E42Q/F66A/T76Y/E81A; E-CRP-2, engineered CRP mutant Y40F/E42Q; moCRP, endogenous mouse CRP; PCh, phosphocholine; WT CRP, native wild-type CRP purified from discarded human body fluids.
